# Complementary expression of EphA7 and SCO-spondin during posterior commissure development

**DOI:** 10.3389/fnana.2014.00049

**Published:** 2014-06-24

**Authors:** Karen Stanic, América Vera, Melissa González, Antonia Recabal, Allison Astuya, Marcela Torrejón, Hernán Montecinos, Teresa Caprile

**Affiliations:** ^1^Axon Guidance Laboratory, Department of Cell Biology, Faculty of Biological Sciences, University of Concepción, Concepción, Chile; ^2^Laboratory of Cell Culture and Marine Genomics, Marine Biotechnology Unit, Faculty of Natural and Oceanographic Sciences and Program COPAS Sur-Austral, University of Concepción, Concepción, Chile; ^3^Department of Biochemistry and Molecular Biology, Faculty of Biological Sciences, University of Concepción, Concepción, Chile

**Keywords:** EphA7, posterior commissure, SCO-spondin, axon guidance, subcommissural organ, chick embryo, prosomere 1

## Abstract

Bilaterally symmetric organisms need to exchange information between the two sides of their bodies in order to integrate sensory inputs and coordinate motor control. This exchange occurs through commissures formed by neurons that project axons across the midline to the contralateral side of the central nervous system. The posterior commissure is the first transversal axonal tract of the embryonic vertebrate brain. It is located in the dorsal portion of the prosomere 1, at the caudal diencephalon. The axons of the posterior commissure principally come from neurons of ventrolateral and dorsolateral pretectal nuclei (parvocellular and magnocellular nucleus of the posterior commissure, respectively) that extend their axons toward the dorsal region. The trajectory of these axons can be divided into the following three stages: (1) dorsal axon extension towards the lateral roof plate; (2) fasciculation in the lateral roof plate; and (3) midline decision of turning to the ipsilateral side or continuing to the opposite side. The mechanisms and molecules that guide the axons during these steps are unknown. In the present work, immunohistochemical and *in situ* hybridization analyses were performed, with results suggesting the participation of EphA7 in guiding axons from the ventral to the dorsal region of the prosomere 1 through the generation of an axonal corridor limited by repulsive EphA7 walls. At the lateral roof plate, the axons became fasciculated in presence of SCO-spondin until reaching the midline. Finally, EphA7 expression was observed in the diencephalic midline roof plate, specifically in the region where some axons turn to the ipsilateral side, suggesting its participation in this decision. In summary, the present work proposes a mechanism of posterior commissure formation orchestrated by the complementary expression of the axon guidance cues SCO-spondin and EphA7.

## INTRODUCTION

The vertebrate central nervous system (CNS) is characterized by a profuse connection between its cells. These connections are mainly formed during development by neurons that extend their axons in order to connect with synaptic counterparts that can sometimes be located at long distances. Axon navigation is controlled by the growth cone, a specialized sensorimotor structure on the leading edge of axons that perceives and interprets different attractive and repellent guidance cues in the environment as axons follow specific trajectories to target areas (Tessier-Lavigne and Goodman, 1996; Mueller, 1999; Dickson, 2002).

A significant advance in the study of axon guidance was the realization that complicated axonal trajectories can be broken down into a series of short segments. Thus, the difficult task of determining how a given axon reaches a distant target is reduced to understanding how the axons navigate between successive segments (Tessier-Lavigne and Goodman, 1996). The end of one segment and the beginning of the next is marked by so-called “intermediate targets,” which consist of small groups of specialized cells that present pathfinding growth cones with the guidance information required to guide the trailing axon along the next segment of its trip (Dickson, 2002).

Bilaterally symmetric organisms need to exchange information between the two sides of their bodies in order to integrate sensory inputs and coordinate motor control. This exchange occurs through commissures formed by neurons that project axons across the midline to the contralateral side of the CNS. Other neurons extend axons that never cross the midline, instead projecting exclusively on their own (ipsilateral) side of the CNS (Dickson and Zou, 2010). Several studies demonstrate that specialized ventral and dorsal midline cells play critical roles in regulating the guidance of both crossing and non-crossing axons by secreting axon guidance cues that attract or repel the growing axons. These cues exist in either diffusible (i.e., netrins, F-spondin, and slit) or cell surface-associated (i.e., semaphorins, Eph, and ephrins) forms that are capable of regulating long- or short-range axonal guidance, respectively (Chilton, 2006). Learning what these guidance cues are and where they are expressed is essential for understanding how the functional brain is constructed. For example, the midline cells in the optic chiasm express ephrin-B2, which induces repulsion to axons that express the EphB1 receptor. In this case, the presence of EphB1 is necessary for generating an ipsilateral response (Nakagawa et al., 2000; Petros et al., 2009).

The mechanisms behind axon guidance have been principally studied in the spinal cord, where there is a subset of cells in the ventral and dorsal midline, floor plate (FP) and roof plate (RP) respectively, that secrete factors involved in axon guidance (Kennedy et al., 1994; Matise et al., 1999; Kaprielian et al., 2001; Dickson and Zou, 2010). In this model, the axons are born in the dorsal region of the spinal cord and extend ventrally towards the FP. Commissural axons are initially repelled by members of the bone morphogenetic protein (BMP) and wingless-related mouse mammary tumor virus integration site (Wnt) families which emanate from the RP and towards the ventral spinal cord, where they are attracted to the FP by combinations of different guidance cues such as Netrin and Shh (Butler and Dodd, 2003; Charron and Tessier-Lavigne, 2005). There are also repellent cues, such as members of the Eph/ephrin family, which prevent ipsilateral axons from midline crossing (Nawabi and Castellani, 2011).

On the encephalic level, the first transversal commissure to develop is the posterior commissure (PC), a conspicuous decussation of fibers originating mainly in the pretectal nuclei and serving auxiliary visual functions (Mastick and Easter, 1996). The PC crosses the midline through the dorsal portion of the prosomere 1, and its caudal border is a landmark for identifying the diencephalic–mesencephalic boundary (Puelles and Rubenstein, 2003; Ferran et al., 2007). The PC axons principally come from neurons of the magnocellular nucleus of the posterior commissure (MCPC) located at the ventrolateral pretectum that run dorsally to the midline and from neurons of the parvocellular nucleus of the posterior commissure (PCPC) that project ventrally in order to reach and cross the midline RP (Ferran et al., 2007, 2009; Merchán et al., 2011; Ware and Schubert, 2011). The molecules involved in guiding the axons of the PC are not yet fully described, but there is some evidence suggesting the participation of the underlying RP (Bach et al., 2003; Hoyo-Becerra et al., 2010; Stanic et al., 2010; Grondona et al., 2012; Vera et al., 2013).

The RP of the caudal diencephalon is formed by radial glial cells. These cells display long basal processes that cross the nerve bundles of the PC and that are attached to the pial membrane (Sterba et al., 1982; Rodriguez et al., 1992, 1998; Caprile et al., 2009). The pretectal RP grows concomitantly with the PC, and the roof of the fully differentiated caudal diencephalon consists almost entirely of the PC and the underlying radial glial cells, otherwise called the subcommissural organ (SCO) due to its location (Rodriguez et al., 1992). The SCO is a phylogenetically ancient and conserved structure that reaches full development during embryonic life. The adult SCO is a circunventricular organ specialized in the secretion of a giant glycoprotein, SCO-spondin, towards the cerebrospinal fluid where it aggregates and forms the Reissner fiber (Rodriguez et al., 1998).

The RP of the pretectum, or embryonic SCO, is divided into two regions, a bilateral diencephalic RP (LDRP), which secretes the SCO-spondin that is responsible for fasciculation and guiding the axons towards the midline, and a medial diencephalic RP (MDRP), where some axons turn to the ipsilateral side in response to unknown guidance cues (Stanic et al., 2010). Given this lack of knowledge, the aim of the present work was to elucidate the guidance cues secreted by the midline cells that are responsible for turning ipsilateral axons. As these axons turn exactly at the midline, the MDRP cells may express a repulsive guidance cue that is probably not diffusible.

The Eph receptor tyrosine kinase family and their ephrin ligands are membrane-bound guidance cues with pleiotropic effects during development, and the repulsive effect of these during axon guidance has been widely studied. The Eph family is subdivided into A and B classes that bind to ephrin-A and ephrin-B ligands, respectively (Gale et al., 1996). All Eph receptors, as well as ephrin-Bs, are transmembrane proteins, whereas ephrin-As are glycophosphatidylinositol (GPI)-linked to the cell membrane. EphAs and ephrin-As can also transduce signals bidirectionally, indicating that ephrin-As have a reverse signal despite the lack of an intracellular domain (Davy et al., 1999; Huai and Drescher, 2001; Davy and Soriano, 2005). Reverse signaling by ephrin-As has been implicated in the pathfinding of vomeronasal (Knoll et al., 2001) and spinal motor axons (Marquardt et al., 2005), the topographic mapping of the axons of olfactory neurons (Cutforth et al., 2003), and retinal ganglion cells (RGCs) (Rashid et al., 2005; Lim et al., 2008).

Previous *in situ* hybridization studies report a dynamic expression pattern of EphA7 during chick brain development (Araujo and Nieto, 1997; Marin et al., 2001; Baker and Antin, 2003; García-Calero et al., 2006). At the level of prosomere 1, the authors describe the presence of EphA7 in the RP and in the alar plate of the commissural and juxtacommisural subdivisions of the prosomere 1 at stage HH13–26 (García-Calero et al., 2006) and HH30 (Marin et al., 2001). This expression occurs concomitantly with the PC development, suggesting that this protein may be involved in the guidance of PC axons.

The present study evaluated the expression of EphA7 during the formation of the PC. For the first time, immunohistochemistry experiments were performed for EphA7 at different stages of development, and its relation to axons of the PC and other guidance cues expressed in the region, such as SCO-spondin, was analyzed. This protein was found to be specifically expressed at the medial diencephalic RP, where axons choose whether to cross or not cross the midline, but not in the bilateral RP. Immunoreaction for EphA7 was also found at the alar plate, forming cellular barriers that enclosed the axons along the ventro-dorsal pathway. The precise location of EphA7 in axonal choice points suggests its role as a guidance cue during PC development.

## MATERIALS AND METHODS

### CHICK EMBRYOS

Fertilized chick eggs were incubated at 38^∘^C in a humidified incubator for specific time intervals. Embryos were staged according to Hamburger and Hamilton (1992; HH stage). In this study, all animals were handled in strict accordance with the Animal Welfare Assurance and followed the guidelines outlined in the Biosafety and Bioethics Manual of the National Commission of Scientific and Technological Research (CONICYT, Chilean Government). All animal work was approved by the appropriate Ethics and Animal Care and Use Committee of the University of Concepción, Chile.

**FIGURE 1:**
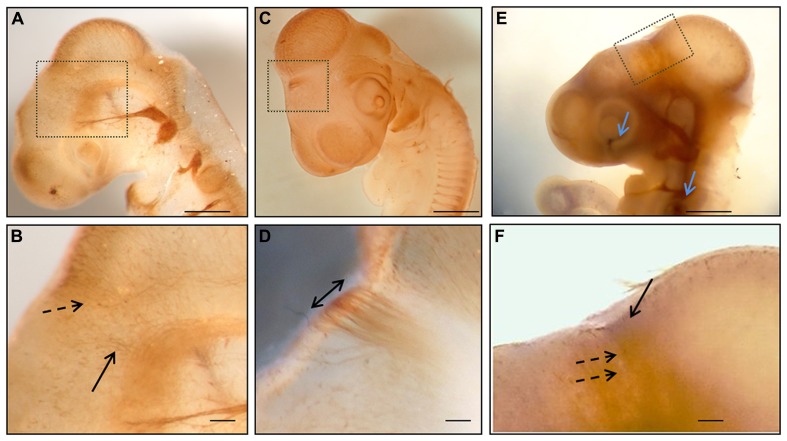
**Whole mount immunohistochemistry during the first stages of posterior commissure development.** **(A,B)** HH17 embryos immunolabeled with 3A10 clone (DSHB) showing axons from neurons of the dorsal area of prosomere 1 (discontinuous arrow in **B**) and from the basal diencephalon (continuous arrow in **B**). **(C,D)** HH18 chick embryo immunostained with anti-tubulin βIII showing the posterior commissure as the first transversal tract on the dorsal encephalon (double arrow in **D**). **(E,F)** HH17 chick embryo immunolabeled with anti-tubulin βIII (brown) and anti-EphA7 (blue) showing the expression of EphA7 (continuous arrow in **F**) in the dorsal diencephalon concomitantly with the axons of the posterior commissure (discontinuous arrows in **F**), as well as in control points such as the retina and otic vesicle (blue arrows in **E**). **(B,D,E)** Magnification of the inset in **(A,C,E)**, respectively. Bars in **(A,C,E)** = 0.5 mm; and in **(B,D,F)** = 0.1 mm.

### *IN SITU* HYBRIDIZATION

#### Generation of the EphA7 riboprobe

Total RNA from the diencephalic RP of HH25 chick embryos and from the whole brain of HH29 chick embryos was purified using RNAqueous (Ambion, Austin, TX). cDNA was synthesized from 1 μg of total RNA using the RevertAid H Minus First Strand cDNA Synthesis Kit (Fermentas, Life Sciences). For PCR amplification, a cDNA aliquot was placed in a total volume of 25 μl containing 2.5 μl of Taq buffer 10×, 1.6 mM of MgCl_2_, 0.4 mM of dNTPs, 0.08 U of Taq DNA polymerase (Fermentas, Life Sciences), and 0.4 mM of primers. This was then incubated at 95^∘^C for 10 min, 95^∘^C for 30 s, 56^∘^C for 60 s, and 72^∘^C for 20 s for 35 cycles with a final elongation step of 5 min at 72^∘^C. Primers were designed based on GenBank chick sequences to analyze the expression of EphA7 (Accession number NM_205083.1), and these were Forward 5′-TTGGCCGATATGGGATATGT-3′; Reverse 5′-GCAAACGATAGCCTTCTTCG-3′ (expected size 322 bp). Amplified products were analyzed by electrophoresis on 1.2% agarose gel, purified with the Qiaquick PCR Purification Kit (Qiagen, Germany), and cloned into the pCR^®^2.1-TOPO^®^ (Invitrogen, Carlsbad, CA, USA) dual promoter vector possessing the Sp6 and T7 promoter sites on each side of the multiple cloning site. One Shot TOP10 *Escherichia coli* (Invitrogen, Carlsbad, CA, USA) were transformed with this vector. Posterior purification of the plasmid was performed with the E.Z.N.A. Plasmid Mini Kit (Omega Bio-Tek Inc., USA) and sequenced (Universidad Católica de Chile) in order to confirm that the cloned PCR product correspond to EphA7 and define its orientation. Digoxigenin (DIG)-11-UTP-labeled single stranded RNA probes (sense and antisense) were prepared with the DIG RNA Labeling Kit using the corresponding mix for SP6 or T7 RNA polymerase (Roche, Germany) according to the manufacturer’s instructions.

#### Whole mount *in situ* hybridization

A modified protocol of Bronner-Fraser and Garcia-Castro (2008) was used. Embryos at different stages of development were collected in ice-cold PBS, fixed with 4% paraformaldehyde/PBS for 2 h, and stored in methanol at –20^∘^C. For analyses, the embryos were rehydrated through a descending series of methanol solutions in PBT (PBS with 0.1% Triton X-100), bleached with 1% hydrogen peroxide in PBT for 1 h, treated with 10 μg/ml of proteinase K (US Biological, Swampscott, MA, USA) for 5 min at room temperature (RT), and treated with 2 mg/ml of glycine on PBT for 5 min. The embryos were re-fixed with 4% paraformaldehyde and washed in PBT. Embryos were hybridized at 60^∘^C for 36–48 h with the antisense or sense (as a control) riboprobe in a hybridization solution (0.28 M of NaCl, 0.001 M of Tris–Base, 0.009 M of Tris–HCl, 0.0034 M of NaH_2_PO_4_, 0.005 M of Na_2_HPO_4_, 50% formamide, 20% dextran sulfate, 5% tRNA, and 2% Denhardt’s solution in H_2_O–DEPC) with continuous rocking. This was followed by subsequent washings in 0.1% sodium citrate (SSC) + 0.1% Triton X-100 at 60^∘^C (SSC 20×: 3 M of NaCl and 0.3 M of NaH_2_C_6_H_5_O_7_) and, posteriorly, in KTBT (50 mM of Tris–HCl pH 7.5, 150 mM of NaCl, and 0.1% Triton X-100) at RT. Later, embryos were blocked with 15% heat-inactivated calf serum (HICS) + 0.7% Blocking Reagent Powder (Boehringer, Germany) in KTBT for 3 h at 4^∘^C. The anti-DIG-alkaline phosphatase (AP), Fab fragments (Roche, Germany) were incubated in a blocking solution (HICS + blocking reagent powder) overnight at 4^∘^C in a dilution of 1:2000. After incubations, the embryos were washed with KTBT eight times for 1 h and rinsed in NTMT (100 mM of Tris–HCl pH 9.5, 50 mM of MgCl_2_, 100 mM of NaCl, 0.1% Triton X-100, and 1 mM of levamisole). Signals were visualized through the reaction of alkaline phosphatase conjugated anti-DIG with the NTB/BCIP solution (Roche, Germany). Finally, the embryos were stored in 50% glycerol–PBS for posterior analysis under a stereo microscope (Olympus, SZ51).

#### *In situ* hybridization in diencephalic sections

HH26 chick brains were fixed with 4% paraformaldehyde/PBS–DEPC overnight, dehydrated in ascending concentrations of alcohols, and embedded in Paraplast. Brains were oriented in order to obtain frontal sections (5–7 μm sections) of the prosomere 1 and mounted in Vectabond treated slices. The sections were rehydrated in decreasing concentrations of ethanol/PBS–DEPC (5 min ethanol 100, 95, 75, 50, 25%) and two posterior washes in PBS–DEPC.

Tissues were treated with 1 μg/ml of proteinase K for 5 min, re-fixed in 4% paraformaldehyde, and acetylated in 1.26% (v/v) triethanolamine, 36 or 19% (v/v) HCl, and 0.26% (v/v) CH_3_COOH in a H_2_O–DEPC solution for 10 min. Afterwards, sections were permeabilized with 1% Triton X-100 in PBS–DEPC for 30 min and pre-hybridized at RT for 4 h with the same hybridization solution as used in the whole mount treatment, but without the riboprobe. Hybridization was performed overnight with the hybridization solution and different dilutions of the EphA7 ripoprobe in a humidity chamber at 65^∘^C. The sections were washed on 5× and 0.2× SSC (SSC 20×: 3 M of NaCl and 0.3 M of sodium citrate pH 7.0) and B1 solution (0.1 M of Tris, pH 7.5, 0.15 M of NaCl, and 0.1% Triton X-100, pH 7.5). This was followed by a blocking step using heat inactivated calf serum (HICF) at 10% with 0.1 M of lysine (Sigma-Aldrich) in B1 solution at RT for 4 h. Once blocked, the tissue was incubated with anti-DIG Fab (Roche, Germany) to a dilution of 1:2000 in B1 solution and 1% HICS in a wet chamber, followed by a wash in B1 solution and equilibration in B2 solution (0.1 M of Tris, pH 9.5, 0.1 M of NaCl, and 50 mM of MgCl_2_ and 1% of Tween 20). Sections were incubated in the dark with NTB/BCIP (Roche, Germany) and analyzed by light microscopy (Leica DME, Leica Microsystems Inc.).

### IMMUNOHISTOCHEMISTRY

Immunohistochemistry was performed as described previously (Stanic et al., 2010). Antibodies were diluted in Tris–HCl buffer containing 1% bovine serum albumin (Tris–BSA).The antibodies used were rabbit anti-EphA7 (Ab5411-100, Abcam, dilution 1/200), mouse anti-βIII tubulin antibody (clone Tuj1, Promega, Madison, WI, USA; dilution 1/1000), 3A10 (Developmental Studies Hybridoma Bank, dilution 1/2), and the anti-Reissner’s fiber glycoproteins antibody that recognizes SCO-spondin made in rabbit or rat (dilution 1/1000; kindly donated by E. Rodriguez; Caprile et al., 2009). For immunofluorescence staining goat anti-mouse Alexa-546 and anti-rabbit Alexa-488 antibodies (Invitrogen, Carlsbad, CA, USA) were diluted 1:100 in Tris–BSA and incubated for 2 h at RT. Nuclei were visualized with TOPRO-3 (Invitrogen). For peroxidase (PO) staining, sections were incubated with a goat anti-mouse or anti-rabbit IgG coupled to PO (Jackson Immunoresearch, West Grove, PA, USA) diluted 1:100 in the same buffer.

For double PO/AP staining, sections were treated with 1 mM of levamisole and 3% H_2_O_2_ to inactivate AP and PO endogenous enzymes. The sections were then incubated overnight with both primary antibodies. Afterwards, the sections were incubated with anti-rabbit IgG coupled with AP and anti-rat or mouse IgG coupled with PO (Jackson Immunoresearch) for 4 h in dilution 1:100 on Tris–BSA. Color development was first performed in AP coupled antibodies through the equilibration of the sections in Tris pH 9.5 and the posterior addition of vector blue solution (Vector Labs, Burlingame, CA) or NTB/BCIP (Roche). The reaction was stopped by washing with the Tris buffer pH 7.8. The PO immunoreaction was visualized with 0.7 mg/ml of diaminobencidine (Sigma) in the presence of 0.001% H_2_O_2_. Sections were mounted in an aqueous Faramount medium (Dako) and visualized by light microscopy (Leica DME, Leica microsystem Inc.). The intensity of the immunoreaction was analyzed with the ImageJ software through transforming the image to a pseudocolor picture, with posterior graphics showing variations in color intensity.

## RESULTS

### PC DEVELOPMENT AND EphA7

The expression pattern of EphA7 was analyzed in relation to the organization of the axons of the PC. Initially, the pathway followed by the PC axons and the expression of EphA7 in this trajectory were examined. It has been previously reported that in the chick embryo this commissure is formed by dorsally projecting axons from neurons located in the ventral pretectum and by axons from neurons located in the dorsal pretectum (Ware and Schubert, 2011). In order to confirm these observations, immunohistochemistry for tubulin βIII was performed *in toto* at different stages of development (**Figure 1**), revealing that at early stages (HH17) axons from the ventral alar plate of the pretectum (continuous arrow in **Figure 1B**), as well as from neurons of the dorsal alar plate (discontinuous arrow in **Figure 1B**), advance towards the pretectal RP in order to conform the PC. At the HH18 stage, the PC was already visible at the first axonal tract of the dorsal region (double arrow in **Figure 1D**). From these results, the expression of EphA7 was analyzed during the incipient formation of the PC via double immunohistochemistry* in toto*, with observations revealing that EphA7 was expressed in the region of the prosomere 1 (continuous arrow in **Figure 1F**), just where the axons advance from the ventral alar plate (discontinuous arrows in **Figure 1F**).

**FIGURE 2:**
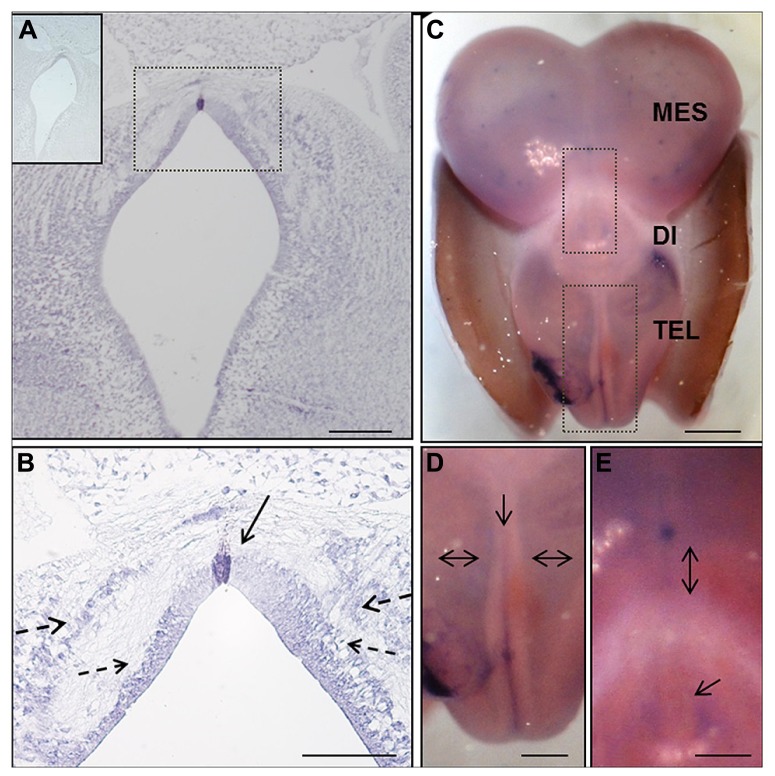
***In situ* hybridization of EphA7 during chick brain development.** **(A,B)** Frontal section of prosomere 1 of the HH25 chick embryo, showing the expression of this transcript in the midline cells of the diencephalic roof plate (continuous arrow in **B**), and in the alar and lateral plates forming two columns parallel to the ventricular wall (discontinuous arrow in **B**). Upper left in **(A)** Sense riboprobe control. **(C–E)** Whole mount *in situ* hybridization of the HH30 embryo brain. **(C)** Expression of the transcript along the encephalon, including the telencephalic midline (arrow in **D**) and alar plate (double arrows in **D**), as well as around the pineal gland (arrow in **E**) and in the diencephalic–mesencephalic boundary (double arrow in **E**). Tel: Telencephalon; Di: Diencephalon; and Mes: Mesencephalon. Bars in **(A)** = 200 μm; **(B)** = 100 μm; **(C)** = 1 mm; and in **(D,E)** = 0.5 mm.

The expression of EphA7 at latter stages of development was analyzed by *in situ* hybridization (**Figure 2**), with results showing that at HH25, EphA7 was heavily expressed in a few cells located at the midline of the RP (continuous arrow in **Figure 2B**) and in cells of the alar plate that were grouped to form two ventro-dorsal cellular columns (discontinuous arrows in **Figure 2B**). The expression of EphA7 at stage HH30 was maintained in the dorsal midline at the mesencephalon, diencephalon (**Figure 2E**), and, especially, at the telencephalon, where there was also an expression at the entire alar plate (**Figure 2D**). All together, these results reveal a specific and dynamic expression pattern of EphA7 during development and its localization at important axonal choice points such as the dorsal midline.

**FIGURE 3:**
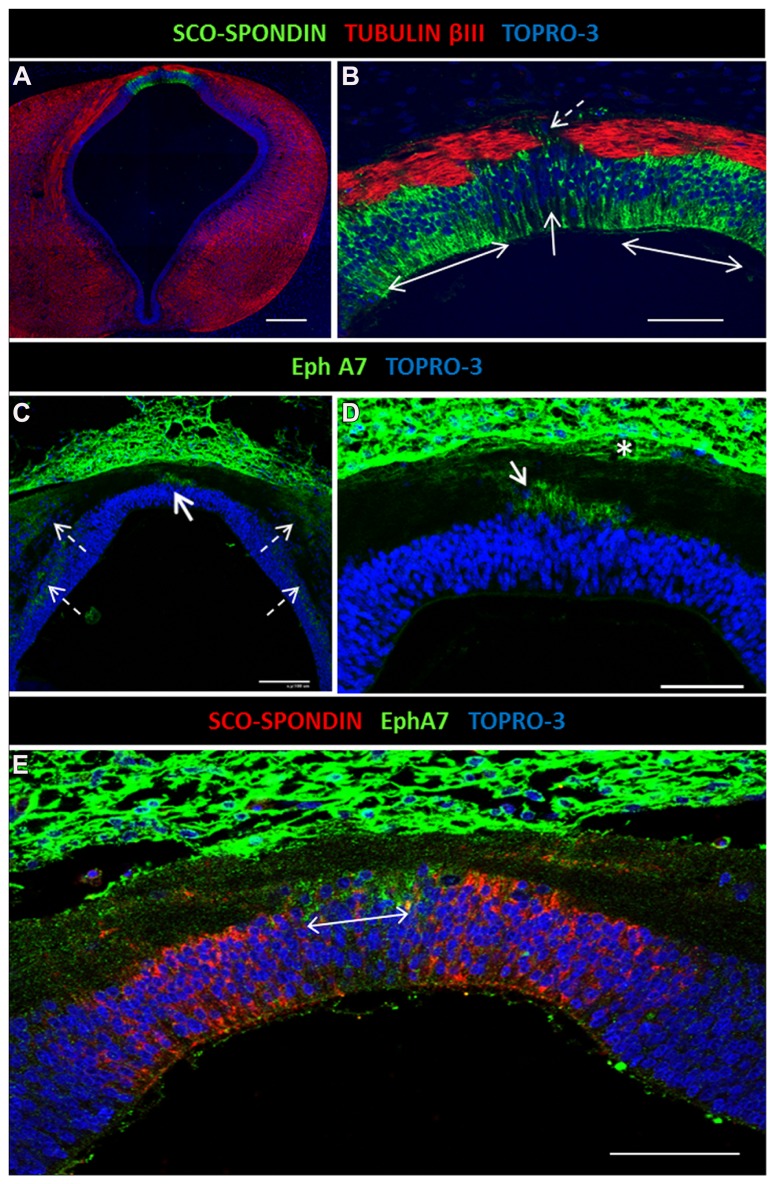
**Expression of EphA7 and SCO-spondin in diencephalic roof plate.** **(A)** Double confocal immunofluorescence in HH30 brain section for SCO-spondin and tubulin βIII showing the expression of SCO-spondin in the lateral diencephalic roof plate (doubles arrows in **B**) and the depletion of this protein in the midline cells (continuous arrow in **B**). The axons change the grade of fasciculation in the midline area, and some turn to the ipsilateral side (discontinuous arrow in **B**). **(C,D)** Expression of EphA7 in the midline cells of the diencephalic roof plate (continuous arrow in **C**) and in longitudinal columns parallel to the ventricular cavity (discontinuous arrows in **C**). At the midline, EphA7 is found near the cell bodies of the roof plate cells (arrow in **D**) and near the pial membrane (asterisk in **D**). **(E)** Double immunohistochemistry for EphA7 and SCO-spondin showing the complementary expression of both proteins in the diencephalic roof plate, with the expression of EphA7 in the midline cells (double arrow) and SCO-spondin in the lateral area. Bars in **(A)** = 200 μm; **(C)** = 100 μm; and in **(B,D,E)** = 50 μm.

**FIGURE 4:**
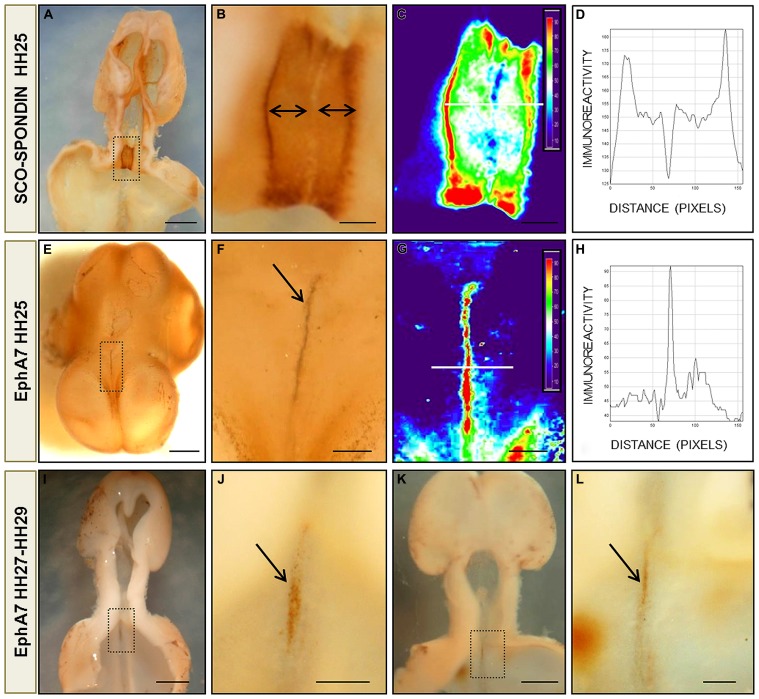
**Protein pattern expression of EphA7 and SCO-spondin during chick brain development.** **(A,B,E,F)** Whole mount immunohistochemistry for SCO-spondin **(A,B)** and EphA7 **(E,F)** of HH25 chick brain embryos showing the complementarity expression between the labeling of SCO-spondin and EphA7 in the diencephalic roof plate (arrows in **B,F**). **(C,G)** Pseudocolor images of **(B,F)** showing the expression level of SCO-spondin and EphA7, respectively. The intensity of the immunoreaction of the white line in **(C,G)** was analyzed in **(D,H)**. **(I,J)** and **(K,L)** Whole mount immunohistochemistry for EphA7 in the diencephalic roof plate in later stages of development (HH27 and HH29, respectively) showing that the expression of EphA7 continues at latter stages but with a lower intensity (arrows in **J,L**). **(B,F,J,L)** Magnification of the inset of **(A,E,I,K)**, respectively. Bars in **(A,E,I,K)** = 1 mm; and in **(B,C,F,G,J,L)** = 0.25 mm.

**FIGURE 5:**
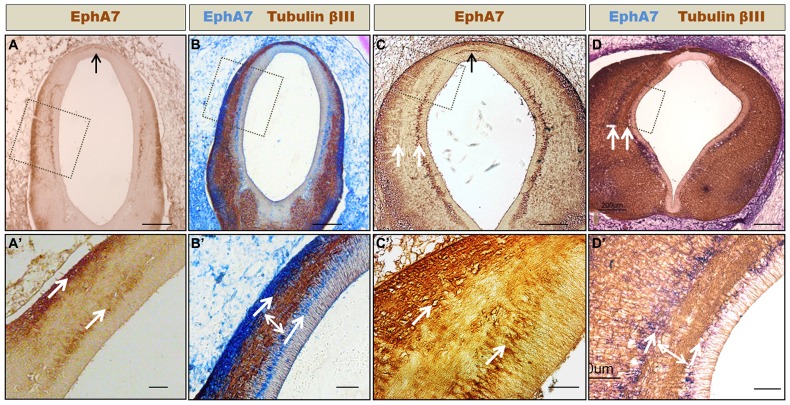
**Formation of axonal corridors delimited by EphA7.** Frontal sections of HH24 **(A,B)** and HH30 **(C,D)** prosomere 1 showing the expression of EphA7 **(A,C)** and EphA7 and tubulin βIII **(B,D)**. At both stages of development, EphA7 maintains the same expression pattern, with localization at the midline cells of the roof plate (black arrows in **A** and **C**) and in two continuous ventro-dorsal columns that traverse the diencephalic wall (white arrows in **C,D,A′–D′**). These columns delimit a corridor where the axons advance towards the dorsal region (double arrow in **B′,D′**). **(A′–D′)** Magnification of the inset of **(A–D)**, respectively. Bars in **(A–D)** = 200 μm; and in **(A′–D′)** = 50 μm.

### COMPLEMENTARY EXPRESSION OF EphA7 AND SCO-SPONDIN

The axon guidance cues responsible for PC formation are not yet fully known. It was previously shown that SCO-spondin is secreted by the lateral diencephalic RP from the early stages of development, but expression was missing in the few midline RP cells (Stanic et al., 2010). *In vivo* and *in vitro* evidence shows that SCO-spondin is important for axon fasciculation, since axons located on the lateral RP are highly fasciculated in contrast to the defasciculated axons located on the midline, where some turn to the ipsilateral side (Stanic et al., 2010; discontinuous arrow in **Figure 3B**). Immunohistochemical analysis of EphA7 expression at the HH30 stage revealed that this protein was expressed at the midline, specifically in cells lacking SCO-spondin (continuous arrows in **Figures 3C,D**; double arrow in **Figure 3E**). The cells of the diencephalic RP presented radial glia morphology, with an apical body in contact with the cerebrospinal fluid and a basal prolongation that traversed the PC and attached to the pial membrane. EphA7 was located in the region of the basal prolongation and was in contact with the axons arriving at the midline. EphA7 was especially expressed in the region near the pial membrane (asterisk in **Figure 3D**) and in the region near the cellular body (arrow in **Figure 3D**). At the immunohistochemical level, expression of EphA7 in the alar plate forming cellular columns was also observed (discontinuous arrows in **Figure 3C**).

On the other hand, the complementary expression of EphA7 and SCO-spondin found by immunohistochemistry in diencephalic slides was confirmed by immunohistochemistry *in toto* at different stages of development. As stated before, SCO-spondin was expressed exclusively in the lateral RP of the prosomere 1, with leaking at a few midline cells (**Figures 4A,B**). Analysis of EphA7 expression between HH25–29 (**Figures 4E–L**) revealed a strong expression at midline dorsal cells. At HH25, expression was circumscribed at the diencephalon (**Figure 4E**), and at latter stages, a weaker signal was also observed in the rostral mesencephalon (**Figures Figures 4I–L**). Pseudocolor analysis (**Figures 4C,G**) and posterior plotting to represent EphA7 and SCO-spondin immunohistochemistry intensity versus distance (**Figures 4D,H**) showed the exact complementarity expression between both guidance cues at the diencephalic RP.

### EphA7 WALLS, LIMITING AXONAL CORRIDORS

As stated before, the axons that form the PC mostly originated from neurons located at the ventral alar plate of the pretectum (MCPC nucleus) and, to a lesser extent, from neurons of the dorsolateral pretectum (PCPC nucleus; **Figure 1B**; Ferran et al., 2009). One of the questions that arose from this was how the axons originating at the ventral pretectum run toward the dorsal region. Immunohistochemical analysis showed that at the pretectal alar plate, EphA7 is arranged in two cellular columns (arrows in **Figure 5**), one near the ependymal wall and the other initially near the pial membrane (**Figure 5A**), which became progressively thicker at later stages (**Figure 5C**). Double immunohistochemistry against tubulin βIII revealed that, enclosed by these EphA7 cellular columns, the axons that formed the PC ran toward the dorsal region (double arrow in **Figures 5B′,D′**).

## DISCUSSION

The present work analyzed the precise localization of EphA7 during diencephalon development and its possible role in the formation of the PC. To achieve these aims, *in situ* hybridization and immunohistochemistry experiments were performed for EphA7 at different stages of development, showing that this protein is present at axonal choice points with a very specific distribution from the beginning of PC formation, thus suggesting its participation in guiding PC axons.

**FIGURE 6:**
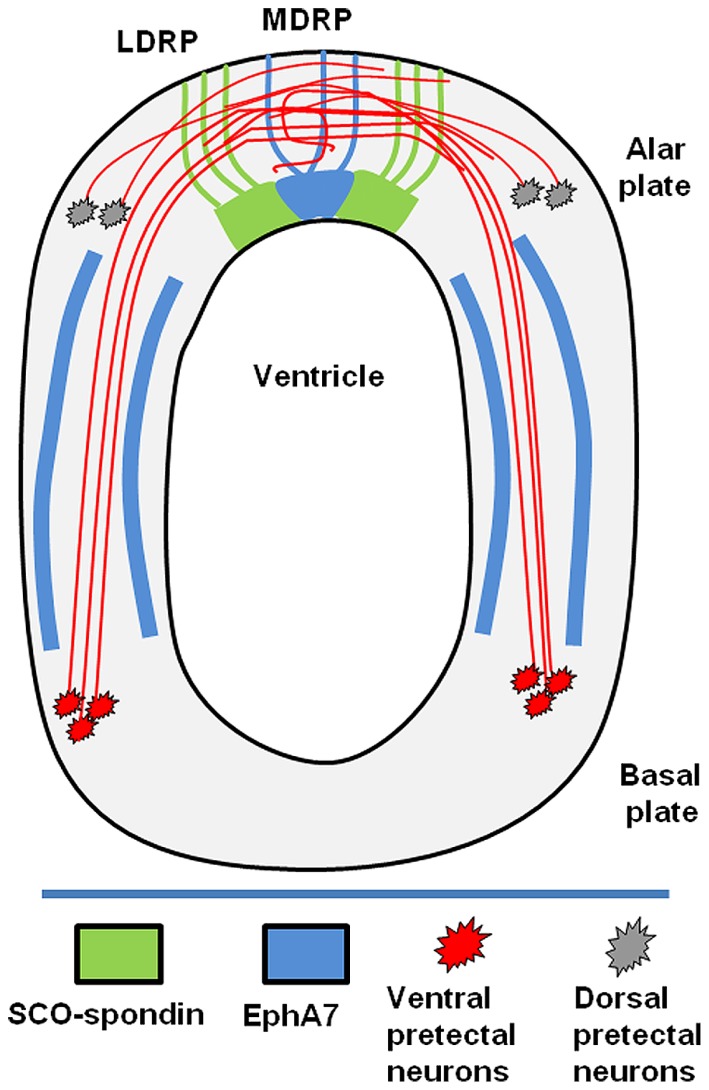
**Schematic drawing showing the diencephalic localization of EphA7.** The posterior commissure is formed by axons from neurons of the basal plate (red) and alar plate (gray). The axons of the basal plate run dorsally inside of corridors delimited by EphA7 (blue). At the lateral roof plate, SCO-spondin (green) promotes the axonal fasciculation until the midline, where in the presence of EphA7; some of the axons turn to the ipsilateral side.

The axons of the PC came from neurons of the ventral pretectal nucleus that extended their axons dorsally and from axons of neurons located in the dorsal pretectum (**Figure 6**). In the LDRP, these axons became fasciculated and advanced towards the MDRP, where some turned to the ipsilateral side (Stanic et al., 2010 and present work **Figure 3**). The trajectory of these axons can be divided into the following three stages (**Figure 6**): (1) advance from the ventral region to the lateral RP; (2) fasciculation in the lateral RP; and (3) midline decision to turn towards the ipsilateral side or continue to the opposite side.

The results obtained in the present work suggest that EphA7 participates in the first and the last of these stages. Initially, the cellular columns of EphA7 in the alar plate delineated an axonal corridor that enclosed the axons until they reached the lateral RP. These results suggest that the EphA7 barriers may provide a repulsive force that constrains the axonal navigation pathway and prevents axons for exiting to the outgrowth corridor. Similar repulsive forces mediated by EphA7 have been described in wild type retinal axons (Rashid et al., 2005; Lim et al., 2008).

At the lateral RP, axons are in contact with SCO-spondin. *In vivo* and *in vitro* evidence suggests that this protein participates in axon growth and fasciculation, forming tunnels that define the axonal route toward the midline, where in the absence of SCO-spondin, axons become defasciculated and face the option of crossing the midline or not (Stanic et al., 2010; Vera et al., 2013).

On the other hand, the precise localization of EphA7 at the dorsal midline, exactly where some axons turn to the ipsilateral side, suggests its participation in this axonal decision. At this locality, EphA7 could have two possible roles. It may participate in the turning of the ipsilateral axons, or it may prevent commissural axons from recrossing the midline. The participation of Eph members in the decision of crossing or not crossing the midline has been reported in other commissures, such as in corticospinal axons (Kullander et al., 2001), the anterior commissure (Henkemeyer et al., 1996), decussating vestibular efferents (Cowan et al., 2000), and the optic chiasm (Williams et al., 2003), where it classically exerts a repulsive effect upon the ipsilateral axons.

The possible participation of EphA7 in the guidance of the axons of the PC opens new questions about how this process occurs. Understanding the function of EphA7 signaling in the embryo requires knowledge not only of the expression pattern, but also of the binding properties of the ligands involved. The presence of some members of the ephrin-A subclass on the prosomere 1 has been reported before. Marin et al. (2001) showed the expression of ephrin-A2 and ephrin-A5 in the pretectal region, with a graded expression of both ephrins from the basal plate to the dorsal plate of the diencephalon and with a high concentration in the principal precommissural nucleus. These observations allow for suggesting that ephrin-A2 and -A5 could be the ligands present in the axons that generate the repulsion effect when in contact with EphA7 of the column walls.

In order to generate intracellular signaling, ephrin-As (GPI-anchored proteins) require interaction with transmembrane co-receptors. It has been reported that ephrin-As interact on RGC axons in *cis* with the p75NTR neurotrophin receptors, which is necessary for the repulsion of ephrin-A-expressing RGC axons from an EphA substrate (Poopalasundaram et al., 2011). Another co-receptor for ephrin-As is TrkB, which increases the axon branch-promoting activity of TrkB (Poopalasundaram et al., 2011). Additionally, it has been recently reported that Ret, a tyrosine kinase protein, is also an ephrin-A co-receptor required for motor axon attraction mediated by ephrin-A reverse signaling. Ret is also capable of binding the soluble ligand GDNF, generating an interrelation between different signaling cues (Bonanomi et al., 2012). In summary, the effect of EphA7 on ephrin-As expressing axons can be repulsion or attraction depending on the co-receptor (P75NTR, Trk, or RET). As stated before, the presence of ephrin-A2 and -A5 in the prosomere 1 has been reported, however, there are no reports about the expression of any of these co-receptors during diencephalon chick development.

Another question that arises from the present results is about the function of EphA7 in RP midline cells. EphA family members are tyrosine kinase receptors, and their stimulation by ephrin-A ligands have been shown to suppress integrin-mediated cell adhesion (Miao et al., 2000; Nakamoto et al., 2004). This could be important since the RP cells and the axons of the posterior commissure possess integrin β1 and integrin α6 (Caprile et al., 2009). Therefore, it is possible that the interaction of EphA7 in the midline cells with its axonal ligand could promote a decrease in cellular adhesion, thus allowing for the turn-off of ipsilateral axons.

In summary, the present study shows that EphA7 presents a dynamic temporo-spatial expression pattern in close contact with the PC axons and with the axon guidance cue SCO-spondin. These results strengthen the idea that EphA7 participates in the development of the posterior commissure, possibly aiding the axons in deciding to cross or not cross the midline. Additionally, the presence of axonal corridors delimited by EphA7 in the pretectal region was observed, representing a new strategy for axon guidance not reported before.

## Conflict of Interest Statement

The authors declare that the research was conducted in the absence of any commercial or financial relationships that could be construed as a potential conflict of interest.
